# Outcome at Two Years of Very Preterm Infants Born after Rupture of Membranes before Viability

**DOI:** 10.1371/journal.pone.0166130

**Published:** 2016-11-09

**Authors:** Amelie Kieffer, Gaelle Pinto Cardoso, Caroline Thill, Eric Verspyck, Stéphane Marret, for the Perinatal network of Haute-Normandie

**Affiliations:** 1 Department of Neonatal Medicine, Functional Education Centre for the Child and Reference Centre for learning disabilities, Rouen University Hospital, Rouen, France; 2 Region-Inserm team (ERI 28) "Neovasc", Microvascular endothelium and perinatal cerebral lesions, Institute for Biomedical Research and Innovation, School of Medicine, Rouen University, Rouen, France; 3 Department of Biostatistics, Rouen University Hospital, Rouen, France; 4 Department of Obstetrics, Rouen University Hospital, Rouen, France; Johns Hopkins University, UNITED STATES

## Abstract

**Purpose:**

To compare the respiratory and neurological outcomes at two years of age of preterm children born before 33 weeks of gestation (WG) after early preterm premature rupture of membranes (EPPROM) between 14 and 24 WG with preterm children without EPPROM.

**Design and Patients:**

This single-center case-control retrospective study was conducted at Rouen University Hospital between 1^st^ January 2000 and 31^st^ December 2010. All the cases with EPPROM born from 26WG to 32WG were included. Each newborn was matched by sex, gestational age (GA) and year of birth to two very preterm children, born without EPPROM. At two years of corrected age, motor and cognitive abilities were assessed by routine score based on the Amiel-Tison and Denver developmental scales.

**Results:**

Ninety-four cases with EPPROM before 24WG have been included. The 31 children born from 26WG to 32WG were matched with 62 controls. The EPPROM group had poorer clinical evaluation at one year for motor (p = 0.003) and cognitive developmental scores (p = 0.016). Neuromotor rehabilitation was performed more often (p = 0.013). However, there was no difference at 2 years of age. Children born after EPPROM were hospitalized more often for bronchiolitis (p<0.001) during their first 2 years, which correlates with increased incidence of pneumothorax (p = 0.017), pulmonary hypoplasia (p = 0.004) and bronchopulmonary dysplasia (p = 0.005) during neonatal period.

**Conclusion:**

At two years, despite an increase in severe bronchiolitis and the need for more neuromotor rehabilitation during the first month of the life after discharge, there was no difference in neurological outcomes in the very preterm children of the EPPROM group compared to those born at a similar GA without EPPROM.

## Introduction

Preterm premature rupture of membranes (PPROM) before 37 weeks gestation (WG) complicates 3% of pregnancies and is involved in 30–40% of preterm births [1]. Currently, there is no consensus on therapeutic approach faced with early preterm premature rupture of membranes (EPPROM) occurring between 14 and 24 WG before viability of the fetus. A common approach is expectant management hoping to reach a viable period from 23-24WG in order to use corticosteroids, antibiotics and transfer to level III ward [2,3,4,5]. Antenatal prognostic factors for neonatal outcomes after PPROM are gestational age at rupture and birth [6], duration of latency before birth [7] and association with oligohydramnios [8], but they are less known for EPPROM. In a previous study, we observed that EPPROM prior to viability was an independent risk factor for neonatal respiratory adverse outcome of preterm children [9]. However, extreme prematurity may represent the main risk factor for all perinatal adverse outcomes, in particular inflammatory diseases such as intraventricular hemorrhages, necrotizing enterocolitis or chronic lung disease [9]. Few studies have focused on the long-term outcomes of infants born after EPPROM. The aim of our study was to compare the respiratory and neurological outcomes at two years of age of very preterm children born before 33WG after EPPROM with very preterm children without EPPROM.

## Methods and Patients

### Selection criteria and procedures

This single-center comparative retrospective study was conducted at Rouen University Hospital between 1st January 2000 and 31st December 2010. All pregnancies with EPPROM between 14^+0^ and 23^+6^ WG were identified from the obstetrical and neonatal database of Rouen University Hospital. They were singleton pregnancies with EPPROM diagnosed from the results of amniotic fluid flow from the endocervix and confirmed by biochemical test (nitrazine/IGFBP-1) when necessary. Gestational age was estimated on the basis of the date of the last menstrual period and early prenatal ultrasound examination, which is routine practice in France. Multiple pregnancies and fetal malformations have been excluded. From the 30452 deliveries identified from the database, we identified 94 cases with EPPROM including 31 children born prematurely before 33WG. We performed a case control study. Each very premature infant born following EPPROM between 14 and 24 WG was matched at birth with two non-malformed babies born after idiopathic preterm labour. The latter was defined as spontaneous onset of labour before rupture of membranes or with rupture of membranes of less than 12 hours duration, according to gestational age at birth (+/- 3 days), sex and birth date (+/- 6 months). Each very preterm infant admitted to the Rouen University Hospital are routinely followed up after discharge. Parents signed a consent for admission in the Perinatal network of Haute-Normandie. The study was approved by the Regional Institutional Ethics Committee of Rouen.

### Medical record review

Maternal data were collected as follows: age, parity, body mass index, gynecological history, obstetrical history, antenatal care and labour supervision with administration of antenatal steroids, tocolysis, fetal heart rate monitoring, caesarean section and its cause, and delivery in a context of chorioamnionitis. Diagnosis of chorioamnionitis was considered in the presence of two or more of the following: maternal temperature >38°5 with absence of other infection, uterine contractions and pelvic tenderness, fetal tachycardia with reduced oscillations, or biological abnormalities (C-reactive protein > 20/mm^3^, white blood cell count > 15 000 mm^-3^).

Neonatal data were also recorded as follows: gestational age at birth, sex, birth weight, duration of rupture of the amniotic sac yolk, five-minute Apgar score, pH at birth, resuscitation in the delivery room, and duration of intubation or Continuous Positive Airway Pressure over 48 hours. Neonatal complications were neonatal respiratory disease, pneumothorax, pulmonary hypoplasia, chronic lung disease defined by oxygen dependency at 36 weeks corrected gestational age, necrotizing enterocolitis (NEC) defined according to Bell's criteria [10], intra-ventricular hemorrhage (IVH) stage III defined by massive IVH associated with ventricular dilatation, and stage IV defined by IVH associated with parenchymal lesions, periventricular leukomalacia (PVL) defined by persisting hyperechogenicities in the white matter at more than 8 days interval or periventricular cavitations in cranial ultrasonographic studies, length of stay in NICU and total duration of hospitalization, and corrected gestational age at the end of hospitalization. Before discharge at home, each infant had had a RSV prophylaxis if he met the requirements of the routine protocol of the NICU: birth weight less than1000 g, preterm infants born before 28 WG, preterm infants born before 30 WG presenting a chronic lung disease at 36 WG. The definition of pulmonary hypoplasia is anatomopathological but we used clinical criteria in our study: a low chest expansion with respiratory distress with ineffective ventilation or with high and prolonged ventilation and often complicated by pulmonary arterial hypertension and pneumothorax.

At 2 years of corrected age (+/- 2 months), we collected data during follow-up consultations: weight, height and head circumference, and trophicity according to WHO curves. Motor and cognitive developments were assessed by routine scores based on the Amiel-Tison and Denver developmental scales [11,12]. These scores are prospectively computed by the paediatricians using a standardized table of evaluation registered in the computer database of the Hospital. For each abnormal score, a severity scale was defined according to specific criteria: normal, moderate or severe impairment.

Normal motor score was defined at age 2 years by walking before age 18 months, protected falls, ability to jump on two feet, run, climb stairs, and stack at least two cubes. Moderate motor impairment was defined at age 2 years by walking after age 18 months, unprotected falls, direct approach to an object, and precision grip. Severe motor impairment was defined at age 2 years by assisted or impossible walk, approximate or missing approach to an object, missing or pathological grip. Cerebral palsy was evaluated according to the European Cerebral Palsy Network definition [13]. Walking age was analysed for each child. The neurorehabilitation is processed by a physical therapist with 2 sessions per week frequency. It starts as soon as an abnormal clinic evaluation is made.

Normal cognitive score was defined as the ability to build a puzzle, get dressed alone, engage in symbolic games, name an image, string two words together, and share with others. Moderate abnormal cognitive impairment at 2 years was defined as show the object on a picture without speaking, speak with isolated words and accept to repeat them, show the different areas of his body, build puzzle randomly, cannot engage in symbolic games. Severe abnormal cognitive impairment was defined by the use of an incomprehensible language without words pronounced correctly, no preference between an object or an activity, an infant that empties the objects of a box and throw them without putting them back in the box, an infant that cannot express his desires with moves or behavior. At extreme impairment level, an infant performs stereotyped activities or sounds and shows an aggressive behavior towards himself or others.

### Statistical analysis

Statistical analysis was performed using SAS version 9.3. A case-control study was performed for maternal data with a logistic regression model matching birth weight, sex and gestational age. For comparison of antenatal care and labour, and neonatal data and outcomes at 2 years of age, a logistic regression model with adjustment for birth weight, sex, and term was performed, with EPPROM as variable. To analyze the difference of walking at two years of age between the two groups, we did a log-Rang test. The significance level was set for p < 0.05.

## Results

**Fig 1:**
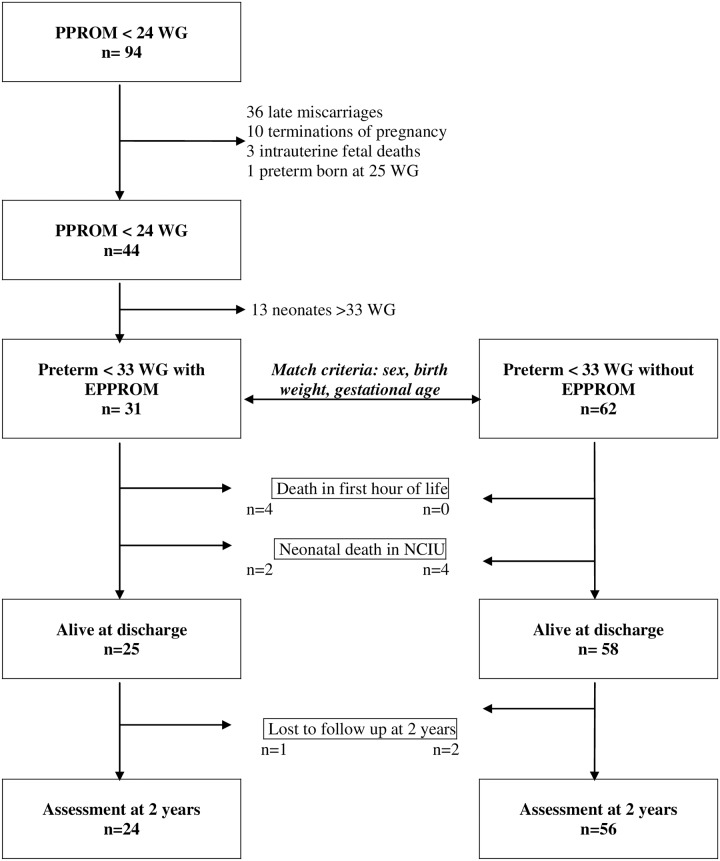
Outcomes of 94 cases of PPROM before 24 WG. WG: Weeks of Gestation; EPPROM: Early Preterm Premature Rupture Of Membranes; NCIU: Neonatal Intensive Care Unit.

Out of the 94 cases of EPPROM, there were 44 (46.8%) live births after 26WG including 31 (33.0%) children born prematurely before 33WG, 1 infant born at 25 WG, 36 (38.3%) cases of late miscarriage (from 14WG to viability), 10 (10.6%) medical terminations of pregnancy, and 3 (3.2%) intrauterine fetal deaths (Fig 1).

The average duration of rupture of the amniotic yolk in the EPPROM group was 51.54 days with a median of 52 days. Duration of rupture extended from 14 to 105 days in the EPPROM group.

The general characteristics of the mothers of each group were compared and are reported in Table 1. The only significant difference was found for the gestity (p = 0.006).

**Table 1:** Comparison of maternal, pregnancy and labour characteristics. SD: standard deviation; ^**1**^ Repetitive urinary or vaginal infections; ^**2**^ Uterus malformation, cervix insufficiency, conisation; ^**3**^ Interruption of pregnancy, C section, preterm birth; * non interpretable.

	Cases n = 31	Controls n = 62	p	OR [CI 95%]
**Maternal age** mean ± SD	28.9 *±* 6.5	28.1 *±* 5.6	0.585	
**Gestity** mean ± SD	2.6 *±* 1.4	1.8 *±* 1.2	0.013	1.6 [1.1–2.3]
**Parity** mean ± SD	2.0 *±* 0.9	1.6 *±* 0.8	0.006	2.0 [1.2–3.4]
**Body mass index** mean ± SD	22.5 *±* 5.9	23.0 *±* 5.5	0.580	
**Infections during pregnancy**^**1**^ n (%)	2 (6.4%)	0	*	*
**Gynecological antecedents**^**2**^ n (%)	1 (3.2%)	2 (3.2%)	*	*
**Obstetrical antecedents**^**3**^ n (%)	10 (32.2%)	24 (38.7%)	0.723	
**Caesarean section antecedents** n (%)	5 (16.1%)	3 (4.8%)	0.084	

Administration of tocolysis was more common in the group without EPPROM (p<0.001). A significant difference was found for antenatal corticosteroids for first treatment (p<0.001) and consolidation (p = 0.005) which were more frequent in the EPPROM group. There was a significant difference regarding gestational age, related to the first course of corticosteroids (p<0.001), administered 15 days earlier in the group of children born in EPPROM context. In contrast, abnormal fetal heart rate (p = 0.007) and use of emergency caesarean section (p = 0.001) were significantly higher in the EPPROM group. The overall results are reported in Table 2.

**Table 2:** Comparison of antenatal care and labour. SD: standard deviation; GA: Gestational Age; WG: weeks of gestation; ^**1**^ Complete cure: 2 injections of corticosteroids at 24H interval; ^**2**^ Renewal of corticosteroid cure 7 days after the first cure; ^**3**^ Diagnosis if at least 2 signs among maternal hyperthermia up to 38°C, maternal or fetal tachycardia, uterine contractions and pelvic tenderness, CRP increase up to 20mg/l; ^**4**^ Difference of means, impact of rupture of membranes on the variable; * non interpretable.

	Cases n = 31	Controls n = 62	p	OR [CI 95%]
**Corticosteroid therapy**^**1**^ n (%)	27 (87.0%)	31 (50.0%)	< 0.001	10.5 [2.7–39.8]
**GA at corticosteroid therapy** mean (WG) (mean days± SD)	25WG+2(177 *±* 6)	26WG+1(183 *±* 15)	< 0.001	-15.4 [-19.1- -11.8]^**4**^
**Consolidation**^**2**^ n (%)	8 (25.8%)	3 (4.8%)	0.005	8.7 [1.9–39.6]
**GA at consolidation** mean (WG) (mean days± SD)	30WG+1(211 *±* 8)	29WG+2(205 *±* 9)	0.187	
**Tocolysis** n (%)	12 (38.7%)	56 (93.0%)	< 0.001	0.02 [0.0–0.1]
**Bradycardia in fetal heart rate monitoring** n (%)	9 (29.0%)	4 (6.2%)	0.007	5.9 [1.6–21.9]
**Caesarean section** n (%)	12 (38.7%)	5 (8.0%)	0.001	7.3 [2.2–23.9]
**Chorioamnionitis**^**3**^ n (%)	10 (32.2%)	0 (0.0%)	*	*

Comparison of immediate neonatal criteria between the two groups is reported in Table 3. Adaptation to extrauterine life was worse in the EPPROM group with a lower 5-minute Apgar (p<0.001) and a higher rate of birth resuscitation.

**Table 3:** Comparison of neonatal criteria. SD: standard deviation; WG: weeks of gestation; gr: gram; ^**1**^ Difference of means, impact of rupture of membranes on the variable; * non interpretable.

	Cases n = 31	Controls n = 62	p	OR [CI 95%]
**Term at birth** mean (WG) ± SD	28.4 ± 2	28.5 ± 2	0.850	
**Birth weight** mean (gr) ± SD	1256 ± 453	1304 ± 362	0.600	
**Sex (male)** n (%)	22/31 (71.0%)	44/62 (71.0%)	1.000	
**Apgar at 5 min** mean ± SD	6.1 ± 2.7	8.3 ± 1.8	< 0.001	-2.2 [-3.1- -1.3]^**1**^
**Cord blood pH** mean ± SD	7.31 ± 0.09	7.30 ± 0.08	0.982	
**External heart massage** n (%)	11/31 (35.5%)	2/62 (3.2%)	*	
**Adrenalin injection** n (%)	5/31 (16.1%)	1/62 (1.6%)	0.018	19.5 [1.6–229.2]
**Intubation in delivery room** n (%)	28/31 (90.3%)	43/62 (69.3%)	0.015	6.8 [1.4–32.0]
**Intubation > 48h** n (%)	11/27 (40.7%)	19/62 (30.6%)	0.310	
**CPAP > 48h** n (%)	20/27 (74.0%)	37/62 (59.7%)	0.140	

Neonatal complications are reported in Table 4. The rates of pneumothorax, pulmonary hypoplasia and chronic lung disease were significantly higher in the EPPROM group than in the control one. The study shows a tendency for later discharge from hospital in children born in the EPPROM context. There was a significant difference in the composite outcome of neonatal death and/or chronic lung disease.

**Table 4:** Comparison of neonatal complications. WG: weeks of gestation; A/S: Affected/Survivors; * Inflammatory disease: Periventricular leukomalacia, and/or IVH and/or chronic lung disease and/or necrotizing enterocolitis and/or death.

	Cases n = 31	Controls n = 62	p	OR [CI 95%]
**Respiratory distress syndrome** A/S (%)	24/27 (88.8%)	56/62 (90.3%)	0.980	
**Pneumothorax** A/S (%)	6/27 (19.3%)	1/62 (1.6%)	0.017	15.2 [1.6–143.3]
**Pulmonary hypoplasia** n (%)	7/31 (22.6%)	1/62 (1.6%)	0.004	27.8 [2.8–278.0]
**Necrotizing enterocolitis** A/S (%)	1/27 (3.7%)	7/62 (11.3%)	0.246	
**III/ IV IVH** A/S (%)	2/27 (7.4%)	2/62 (3.2%)	0.385	
**Periventricular leukomalacia** A/S (%)	1/27 (3.7%)	6/62 (9.7%)	0.396	
**Chronic lung disease** A/S (%)	14/25 (56.0%)	16/58 (27.6%)	0.005	6.9[1.7–26.6]
**NICU duration** mean (days)	14.3	10.5	0.177	
**Hospitalisation duration** mean (days)	75.4	66.4	0.192	
**Age at discharge** mean (WG)	39WG + 4	38WG + 1	0.067	1.4 [0.1–2.9]
**Deaths** n (%)	6 (19.3%)	4 (6.45%)	0.062	3.9 [0.9–16.6]
**Death and/or Chronic lung disease** n (%)	20 (64.5%)	20 (32.2%)	0.002	7.0 [2.0–24.2]
Inflammatory disease* n (%)	10 (32.3%)	19 (30.6%)	0.957	

No significant difference was found between the two groups concerning neonatal non-respiratory complications, nor the combined endpoint of morbidity and mortality with periventricular leukomalacia and/or IVH, and/or enterocolitis, and/or death.

In the EPPROM group, six children died. Four children (one born at 26 WG, two at 27 WG and one at 28 WG) died in the delivery room with severe acute respiratory distress. One child born at 27 WG died at eight days in a context of hypoxic-ischemic encephalopathy, and one child born at 27 WG after fifty-one days of life secondary to necrotizing enterocolitis. In the group without EPPROM, four children died: two children born at 26 WG with severe septic shock at day 11 and day 13, one infant born at 26 WG with pulmonary hemorrhage at day 6, and the fourth child born at 30 WG with necrotizing enterocolitis at day 32.

At two years of age, no additional deaths were reported. Nevertheless, three children were lost to follow-up at two years (one child in the EPPROM group, and two infants in the group without EPPROM). Comparison of outcomes at two years of age is reported in Table 5. There was a significant difference regarding motor and cognitive scores at one year of age, with a higher risk of moderate or severe impairments and neuromotor rehabilitation performed more frequently (p = 0.013) in the EPPROM group. However, at two years of age, no significant difference was identified regarding either motor and cognitive impairments or for walking age. The median age of walking was 18 months in the EPPROM group and 17 months in the control group. Walking was not acquired at two years of age in five children with cerebral palsy in the group without EPPROM (p = 0.861 and Hazard ratio = 1.04 [0.64; 1.70]). There was no difference between the two groups regarding the number of cases of bronchiolitis during the first two years of life, but the number of re-admission to hospital was significantly higher in the case than in the control group (p<0.001).

**Table 5:** Outcome comparisons between both groups at 2 years of age.

	Cases n = 24	Controls n = 56	p	OR [CI 95%]
**Neurological outcome**				
Motor impairment at 1 year (%)	18 (75%)	22 (39.3%)	0.003	7.4 [1.9–28.5]
Cognitive impairment at 1 year (%)	15 (62.5%)	18 (32.1%)	0.016	3.8 [1.3–11.5]
Motor impairment at 2 years (%)	9 (37.5%)	17 (30.3%)	0.592	1.3 [0.4–3.9]
Cognitive impairment at 2 years (%)	10 (41.6%)	17 (30.3%)	0.378	1.6 [0.5–4.6]
Walking independently at 2 years	24 (100%)	51 (91.1%)	0.861	
Mean (months)	18.1	17.2		
Median (months)	18	17		
Neurorehabilitation (%)	16 (66.6%)	20 (35.7%)	0.013	4.3 [1.4–13.6]
Cerebral palsy at 2 years	0	5 (9%)	*	
**Respiratory outcome**				
Number of bronchiolitis *mean ± SD*	0.9 ± 1.5	1.3 ± 2.1	0.572	1.3 [0.5–3.3]
Number of hospitalization *mean ± SD*	2.8 ± 3.4	0.7 ± 1.5	<0.001	2.1 [1.1–3.2]

* non interpretable

Regarding sensorial outcomes, only one child was subject to deafness with aids within control group, no child was subject to visual acuity less than 3/10 at one eye at least in both groups. Results for sensorial outcomes were not interpretable.

## Discussion

In our single-center comparative study, the number of re-admission for respiratory problems remained significantly higher in the EPPROM group than in the control group at two years of age. No difference in neurological developmental outcomes were observed between both groups. However, at one year of age, significant abnormal motor and cognitive abilities and a higher rate of neurorehabilitation needs have been observed in the EPPROM group compared to the control one.

Concerning respiratory outcomes, we observed that EPPROM is an additional factor aggravating the respiratory outcome of very preterm children. Immediate neonatal respiratory adaptation at birth as well as neonatal respiratory diseases were significantly more frequent in the EPPROM group than in the control group, with poorer adaptation to extrauterine life, pneumothorax (19.3% vs 1.6%), lung hypoplasia (22.6% vs 1.6%) and chronic lung disease (56% vs 27.6%). These results corroborate published studies in very preterm infants born in PPROM or EPPROM contexts [14]. At two years of age, although there was no significant difference between the two groups in the number of cases of bronchiolitis, children from the EPPROM group were re-admitted to hospital more often than controls (2.8 vs 0.7), even if most of them had had RSV prophylaxis before discharge. This suggests that chronic inflammatory respiratory diseases are more severe taking their origins during ante- and neonatal periods and suggesting that that lung development have been altered by EPPROM. This hypothesis is further strengthened by the increased trend identified in our study to immediate neonatal mortality after birth (19.3% vs 6.45%), due to bad adaptation to extra-uterine life [15,16,17] and by the significant difference in the combined criteria of death and/or chronic lung disease. These differences are likely partially related to pulmonary hypoplasia, which remains a major factor limiting survival and respiratory adaptation at birth [5]. In the neonatal period, pulmonary hypoplasia is clinically suspected in recurrent pneumothorax, with difficulties to manage assisted ventilation in this specific context of EPPROM and often associated with persistent pulmonary hypertension. However, only histologic post-mortem examination can confirm diagnosis and is not always contributory. Indeed, severity of pulmonary hypoplasia is associated with quantitative and qualitative abnormalities of the lungs, which have not reached expected growth due to oligohydramnios and alteration of lung fluid secretion [18]. In the antenatal period, pulmonary hypoplasia remains very difficult to predict [19]. The risk of pulmonary hypoplasia decreases after 23 WG. It is more related to gestational age at rupture rather than to a long latency period [20,21]. In our study, the rate of chorioamnionitis was higher in EPPROM than in the control group (32.2% vs. 0%) although the result is not statistically significant, due to convergence issue. Chorioamnionitis is considered as an inflammatory and/or infectious disease and is associated with increased risk in neonatal diseases (i.e. necrotizing enterocolitis, periventricular leukomalacia, and chronic lung disease, IVH) [22,23]. But, in our study, except for chronic lung disease and respiratory sequellae, no significant differences were observed between the two groups regarding either neonatal complications, or even a combination of these factors. These results corroborate those of Johanzon [24], who did not find any difference between the groups with PPROM before 28 WG and the group without PPROM for an inflammatory criterion that took into account IVH (stages III and IV), periventricular leukomalacia, necrotizing enterocolitis, chronic lung disease and retinopathy of prematurity (stages III and IV). Moreover, in our study, the rates of cerebral palsy, motor and cognitive impairments were not different at two years of age. This absence of difference in neurodisability rates between the two groups could be explained by the lack of statistical power of a small sample, by the decrease of inflammation thanks to antenatal corticosteroid therapy (more frequent in the EPPROM than in the control group), or by the pre-conditioning to post-natal inflammation induced by fetal inflammatory response syndrome [25]. Antenatal steroids, widely used in EPPROM contexts, have been demonstrated to reduce IVH and cystic periventricular leukomalacia, which are frequently associated with subsequent cerebral palsy [26]. Foix-L'Hélias *et al*. [27] showed in EPIPAGE 1 study that administration of corticosteroids to women at risk of preterm birth had a benefit on the occurrence of periventricular leukomalacia and severe white matter damage in the group of 28-32WG, but not on long-term neurodisabilities at five years of age. Tolerance to post-natal inflammation might also be induced by the immune response of the fetus to intrauterine inflammation after EPPROM. Indeed, the pathophysiological cause of lung injury and neurological sequelae remains largely unknown. Nevertheless, in our study, neurological outcomes at two years of age are encouraging as we observed no difference between EPPROM and control groups. Rib established in 1993 a study showing 72% of surviving infants without impairment at 2 years of age after EPPROM [28]. The retrospective study of Pristauz in 2007 included 87 pregnancies with EPPROM. Out of the 12 survivor infants, half of them had a normal psychomotor development at 2 years of age [29]. These studies were based on small case groups without control. Our study suggests that there is no additional neurological risk with EPPROM compared to gestational age at delivery. We must modulate this comment as we previously noted differences in neurological outcomes at one year of age. There is a higher incidence of neuromotor abnormalities requiring special care in EPPROM infants than controls. Even if these differences were not observed at two years of age, several published studies have evidenced transient neuromotor abnormalities as predicting later cognitive and learning disabilities at school. In EPIPAGE 1 study in 1997, Arnaud [30] showed that the presence of minor transient neuromotor abnormalities at 1 year of age, representing about 40% of preterm infants, had no negative prognostic value on motor levels but was associated with a greater risk of neurocognitive sequelae. Further studies with longer follow up are warranted to confirm or infirm the absence of difference in cognitive deficiencies and learning disabilities at school. Moreover, Manuk showed in a multicenter controlled randomized study that children affected by EPPROM had a poorer outcome at 2 years compared with children affected by PPROM [31].

Our study has some limitations in the fact it is a retrospective study using prospective data collection obtained from a single level III center. EPPROM before viability is a rare event, and it is likely that our study lacks statistical power due to small numbers. At our university hospital, intrauterine transfer is only accepted from 24-25WG. Pairing one exposed child to two unexposed children partly overcomes this pitfall. The database and outcome questionnaires were standardized in a homogenous team of obstetricians and neonatologists, who performed follow-up.

## Conclusion

Few studies have focused on the outcome of very preterm children born following prolonged EPPROM before viability. The results of our study show that gestational age at birth remains the major prognostic factor in this context, even if the rate of mortality and respiratory morbidity is higher than in the control group. In consequence, it is important to give a chance to the fetus affected by EPPROM and not propose systematic termination of pregnancy. Long-term monitoring, including cognitive assessment, remains essential given the high rate of chronic lung disease at 36 WG and neuromotor abnormalities in the first year of life, both of which are associated with greater risk of neurocognitive sequelae.
